# Hyaluronic acid promotes biomineralization of osteoblast-like cells – observations on two different barrier membranes

**DOI:** 10.1186/s40729-025-00646-2

**Published:** 2025-09-08

**Authors:** Daniel Diehl, Vincent Kabst, Tim Bürgel, Thomas Dittmar, Hagen S. Bachmann, Anton Pembaur, Anton Friedmann

**Affiliations:** 1 Department of Periodontology, Center for Biomedical Education and Research (ZBAF), School of Dentistry, Faculty of Health, Witten/Herdecke University, Witten, Germany; 2 Institute of Pharmacology and Toxicology, Center for Biomedical Education and Research (ZBAF), Faculty of Health, Witten/Herdecke University, Witten, Germany; 3 Immunology and Tumor Biology, Center for Biomedical Education and Research (ZBAF), Faculty of Health, Witten/Herdecke University, Witten, Germany; 4 Institute for Clinical Molecular Genetics and Epigenetics, Center for Biomedical Education and Research (ZBAF), Faculty of Health, Witten/Herdecke University, Witten, Germany

**Keywords:** BDDE-crosslinked hyaluronic acid, Guided bone regeneration, Collagen membranes, SaOS-2, Osteogenesis, Alkaline phosphatase, Transcriptome

## Abstract

**Background:**

Guided bone regeneration (GBR) relies on biocompatible membranes to support osteogenesis. 1,4-butanediol diglycidyl ether (BDDE)-crosslinked hyaluronic acid (xHyA) has shown promise in enhancing bone regeneration, yet its mechanisms remain unclear.

**Objective:**

This study evaluates the osteogenic effects of xHyA-functionalized native pericardium collagen membrane (NPCM) and ribose-crosslinked collagen membrane (RCCM) using an airlift culture model with SaOS-2 cells. Transcriptomic changes following xHyA treatment were also investigated.

**Methods:**

SaOS-2 cells were cultured on NPCM and RCCM, with or without xHyA functionalization. Cytocompatibility, alkaline phosphatase (ALP) activity, mineralization (Von Kossa staining), and RNA sequencing were assessed. Differential gene expression and pathway enrichment analyses were conducted on cells exposed to two xHyA concentrations.

**Results:**

Both membrane types supported cell viability, though NPCM allowed cellular infiltration while RCCM maintained barrier integrity. xHyA significantly enhanced ALP activity and mineral deposition across both substrates. RNA sequencing revealed minimal upregulation of classical osteogenic genes but identified differential expression in pathways related to focal adhesion, VEGF signaling, and stem cell differentiation. IGF1R, FYN, and MAPK14 were consistently upregulated regardless of xHyA concentration.

**Conclusion:**

xHyA functionalization enhances osteogenic activity, evidenced by increased ALP and mineralization, likely via modulation of cell-matrix interactions rather than classical osteogenic gene activation. These findings support using xHyA-functionalized membranes in GBR and warrant further investigation in vivo.

## Significance

**What is known**:

• Crosslinked hyaluronic acid (xHyA) enhances cell adhesion, proliferation, and mineralization in bone regeneration models.

• Functionalization of collagen-based GBR membranes with xHyA has shown promise in improving bone formation.

• Limited information is available on the transcriptomic effects of xHyA on osteoblast-like cells.

**What this study adds**:

• This study shows that xHyA-functionalized collagen membranes increase ALP activity and mineral deposition in SaOS-2 cells.

• Transcriptomic analysis reveals xHyA modulates genes linked to cell adhesion and signaling pathways, rather than directly upregulating osteogenic genes.

## Introduction

Guided bone regeneration (GBR) represents a cornerstone of modern oral implantology, widely recognized as an effective technique for local bone augmentation and defect repair. Central to this approach is the use of membranes, which provide a physical barrier to prevent soft tissue ingrowth and support the recruitment, proliferation and differentiation of osteogenic cells at the defect site. The clinical success of GBR relies on membranes that exhibit biocompatibility, appropriate degradation kinetic, and optimized mechanical properties [1, 2]. A crucial property for membrane biocompatibility is the permeability to extracellular fluid, apart from its surface ability to facilitate cell adhesion. These key features contribute to stabilizing the membrane, promote its integration into surrounding tissues, and, for resorbable membranes, enable their degradation.

In recent years, the biofunctionalization of these membranes and grafting materials with bioactive substances has garnered interest for their potential to enhance bone regeneration outcomes [3, 4]. Hyaluronic acid (HA), a naturally occurring glycosaminoglycan, has demonstrated considerable promise in promoting bone regeneration due to its involvement in cell adhesion, proliferation, and differentiation [5]. Crosslinked derivatives of HA, such as 1,4-butanediol diglycidyl ether (BDDE)-crosslinked hyaluronic acid (xHyA), offer increased stability and functionality, making them attractive candidates for tissue engineering applications [6, 7]. Recent studies have highlighted the potential of xHyA to enhance osteogenic differentiation and mineralization, yet the underlying mechanisms remain incompletely understood [6, 8, 9]. Moreover, in vitro experiments by Zhu et al. revealed distinct activation profiles in monocytes after stimulation with low molecular weight HA, high molecular weight HA or a crosslinked modification (xHyA) [10].

Histomorphometric analysis demonstrated that the clinical application of xHyA in GBR yields a higher rate of bone formation and reduced amount of residual xenograft in core biopsies. Also, the composite graft facilitates handling during placement, due to the high viscosity xHya [9, 11]. Recently, various reports have highlighted the regenerative properties of xHya in vitro for cementoblasts and periodontal ligament fibroblasts—two cell types that are highly abundant in the periodontal apparatus. The studies demonstrated a significant increase in alkaline phosphatase and type-1 collagen expression, which are important markers of osteogenicity [12, 13]. While the osteogenic effects of xHya on mesenchymal stem cells have been reported repeatedly, the data regarding the effect of xHya on osteoblasts, particularly their transcriptome, is surprisingly scarce [14].

Therefore, this study investigates the osteogenic effect of xHya functionalization on two porcine collagen-based membranes — a native pericardium collagen membrane (NPCM) and a ribose-crosslinked collagen membrane (RCCM) — using an airlift model with the osteoblast-like SaOS-2 cell line. Moreover, the transcriptome of xHya-treated osteoblast-like cells is investigated. The airlift model allows for simultaneous evaluation of cytocompatibility, mineralization, and gene expression under physiologically relevant conditions. SaOS-2 cells, characterized by their osteoblastic phenotype, serve as a reliable model for studying bone-related processes, although their cancerous origin necessitates careful interpretation of osteogenic marker expression [15, 16].

## Methods

### Cell culture

**Fig. 1:**
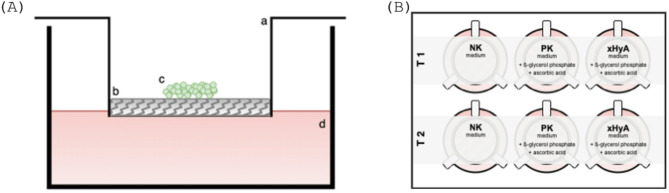
(**A**) Experimental setup of the airlift model. The culture insert (a) is used to carry the substrate (b) and the cells (c) and hold it on the interface between air and medium (d). (**B**) Experimental plate setup. NK = negative control, PK = positive control, xHya = PK medium supplemented with xHya

The osteoblast-like SaOS-2 osteosarcoma cell line (ACC 243, DSMZ, Berlin) was cultivated in Dulbecco’s modified Eagle medium (DMEM, PAN-Biotech) supplemented with 15% (v/v) fetal bovine serum (PAN-Biotech) and 1% (v/v) penicillin-streptomycin (PAN-Biotech). Cells were propagated in a humidified environment (37 °C; 5% CO_2_). The culture medium was renewed every 48 h. Cells from passages 10–20 were used for the experiments. For osteogenic differentiation, the corresponding samples - positive control (PK) and test group (xHyA) - were incubated with an osteogenic differentiation medium consisting of supplemented DMEM + 50 µM L-ascorbic acid-2-phosphate (AA, Carl Roth, Karlsruhe) + 10 mM β-glycerol phosphate (β-GP, Merck, Darmstadt) [17]. For further experiments, cells were seeded onto an airlift model [15]. In brief, 2 × 10^5^ cells were seeded on cell culture inserts (Greiner, 6-well, 8 μm pore size) with an interspersed substrate. Inserts were placed into 6-well plates (Sarstedt) containing enough medium (1.5 ml) to place the substrate on the interface of medium (bottom) and air (top) (Fig. 1). Two substrates were used – a ribose cross-linked collagen membrane (RCCM, Ossix Plus, Regedent AG, Zurich) and a porcine native pericardium membrane (NPCM, Smartbrane, Regedent AG, Zurich). The substrates of the xHya group were pretreated with a BDDE-crosslinked hyaluronic acid formulation (Hyadent BG, Regedent), diluted in phosphate-buffered saline (PBS, 1:100, PAN-Biotech) for 5 minutes. Consequently, a negative control (medium only), a positive control (osteogenic medium), and a test group (xHyA treatment + osteogenic medium) were assayed on a 6-well plate for two to eight days. A total of three biological replicates were performed. For RNA-seq analysis, a total of 3 × 10^6^ cells were seeded in 10 cm cell culture dishes (Sarstedt) and treated with either osteogenic medium or osteogenic medium supplemented with different dilutions of xHya (1:10 and 1:100) for 48 h.

### Alkaline phosphatase assay

Every 48 h, 100 µl of medium was removed from the airlift model to measure alkaline phosphatase activity. In brief, 50 µl of the sample was incubated with 50 µl of p-nitrophenyl phosphate (pNPP, 50mM in acidic buffer, pH 5.5, AAT Bioquest, Pleasanton, CA, USA) for 30 min in the dark. The increase in absorbance at 400 nm was determined using a multi-plate reader (Tecan Infinite200 Pro, Tecan Group AG, Männedorf, Switzerland) at four points per well in a square array. A 100 mU/ml alkaline phosphatase standard (AAT Bioquest) was serially diluted (1:10 in H_2_O + 0.1% BSA) and a standard curve was created by linear regression. The measured value of the blank sample was subtracted from the measured absorbance values of all other samples.

### Viability assay

Cell viability on the NPCM and RCCM substrates was assessed with a water-soluble tetrazolium-8 (WST-8) assay (CCK-8, Abcam, Cambridge, UK) according to the manufacturer’s instructions. In brief, 2 × 10^5^ cells were seeded on the substrate-fitted 6-well culture inserts according to the abovementioned conditions. For the assay, cell culture inserts were submerged in another well on a 6-well plate filled with phenol-red-free medium, supplemented with 10% WST-8 and incubated for 3 h (37 °C). After that, 100 µl of supernatant was transferred to a 96-well plate and assayed on a Tecan multi-plate reader (450 nm).

### Histochemical methods

The NPCM and RCCM substrates were fixed with 70% ethanol for two hours, stained with a 1% aqueous toluidine blue stain, and mounted on a microscope slide (DWK Life Sciences, Wertheim, Germany). Immediately after drying, the substrates were covered with xylene and covering medium (Eukitt, Orsatec, Bobingen, Germany) and examined under a light microscope (Fritz, Precipoint Scan, München, Germany – 60x objective). Further exemplary samples of NPCM and RCCM were fixed by means of glutaraldehyde and sodium cacodylate buffer and osmicated to visualize SaOS-2 cells under the reflected light microscope (Wild M3Z, Leica, Wetzlar, Germany) after eight days of incubation in the air-lift model. Substrates undergoing histological staining were fixed with 70% ethanol for two hours, embedded in kerosene and cut in the middle of the cross-section of the respective collagen membrane. This was followed by melting at 50–60 °C in a heating cabinet for 30 min and the deparaffinization series. Azan staining was chosen as the histological overview staining to highlight the SaOS-2 cells, their cellular components, and the collagen structure of the NPCM and RCCM. Moreover, samples were stained using trichrome, consisting of azocarmine, aniline blue and orange G. Alcian blue staining visualized acidic mucins and sulfated glycosaminoglycans precisely as well as Von Kossa staining was employed as a standard method of staining bone and mineralization in cell culture by detecting calcium.

### RNA isolation and cDNA synthesis

RNA was isolated using the Quick-RNA MiniPrep Kit (R1055, Zymo Research, Freiburg, Germany) according to the manufacturer’s instructions. For reverse transcription, a total of 300 ng RNA was diluted with H_2_O to a volume of 10 µl and mixed with 1 µl of cDNA RT Adapter and 1 µl Annealing Buffer (both SQK-PCS114, Oxford Nanopore Technologies (ONT), Oxford, UK) and ligated.

at 60 °C for 5 min. Next, samples were cooled to room temperature and brought to a volume of 18 µl with 3.6 µl NEBNext Quick Ligation Reaction Buffer (NEB, Ipswich, MA, USA), 1.4 µl T4 DNA Ligase 2 M U/ml (NEB) and 1 µl RNaseOUT (ONT).

Subsequently, 1 µl of lambda exonuclease and 1 µl uracil-specific excision reagent (both ONT) were added to each PCR tube and incubated in the thermal cycler for 15 min at 37 °C. All samples were then transferred to fresh 1.5 ml tubes and mixed with 36 µl RNase-free XP beads (Beckmann-Coulter, Brea, California), washed twice on a magnetic rack and then eluated in 12 µl H_2_O. Finally, 1 µl of RT primers, 1 µl dNTPs, 1 µl reverse transcriptase (Maxima H Minus RT, Thermo Fisher Scientific, Waltham, Massachusetts), and 1 µl of Strand Switching Primer II (ONT) were added, and reverse transcription was performed for 90 min at 42 °C.

### Library preparation

To each sample, 0.75 µl of a unique barcode primer pair, 6.75 µl nuclease-free water, 12.5 µl of 2x LongAmp Hot Start Taq Master Mix (NEB) and then amplified in a PCR cycler. Subsequently, the samples were incubated with 20 µl resuspended AMPure XP beads (Beckman-Coulter) and washed on a magnetic rack with 70% ethanol. The pellets were resuspended in 12 µl elution buffer (ONT). Lastly, 1.2 µl Rapid Adapter T and 6.8 µl RAP-Dilution Buffer (both ONT) were added. The flow cell (R10.4.1, ONT) was loaded with 75 µl of a mixture of 37.5 µl Sequencing Buffer II, 25.5 µl Loading Beads II and 12 µl DNA library and sequenced on the MinION Mk1C nanopore sequencer (ONT).

For this 5 × 10^6^ SaOS-2 cells were seeded in 15 cm dishes. After 24 h (37 °C, 5% CO_2_) the confluent cells were incubated with 15 ml osteogenic medium (OM) (10 mM β-GP and 500 µM ascorbic acid) or with OM with 20 µM Lonafarnib for 72 h (37 °C, 5% CO_2_). Afterwards, the RNA was isolated. For sequencing, the FLO-MIN106D Spot-On Flow Cell R9 Version was used, and the number of viable pores was tested before use. The Tecan multi-plate reader checked the cDNA library for quantity and quality (Tecan Group, Männedorf, Switzerland). For sequencing, the samples were prepared with a Native Barcoding Kit 24 V14 (SQK-NBC111.24, ONT). 200 ng RNA of treated and untreated samples were used in biological replicates and treated as the manufacturers’ protocol indicated. The sequencing library was loaded into the flow cell, and a 24 h sequencing protocol was run.

### Statistical analysis

For the ALP assay, a Repeated Measures ANOVA with a consecutive *post hoc* Tukey multiple comparisons test (α = 0.05) was performed. Raw sequencing data was basecalled in real-time using the integrated Guppy software (v6.5.7) of the MinION Mk1C. The Epi2me labs workflow wf-transcriptomes (v1.1.1) was used for alignment, differential gene expression analysis, and differential transcript usage. Salmon (v1.10.2) was used to assign reads to individual annotated isoforms defined by the GTF-format annotation. These counts were used to perform statistical analysis to identify the genes and isoforms showing abundance differences between the experimental conditions. Statistical analysis was performed using EdgeR to identify the subset of differentially expressed genes using the gene counts as input. The normalization factor was calculated for each sequence using the default TMM method. The differentially expressed genes were corrected for false discovery (FDR) using Benjamini and Hochberg’s method. Differential transcript usage was performed using the R DEXSeq package (v1.46.0).

## Results

**Fig. 2:**
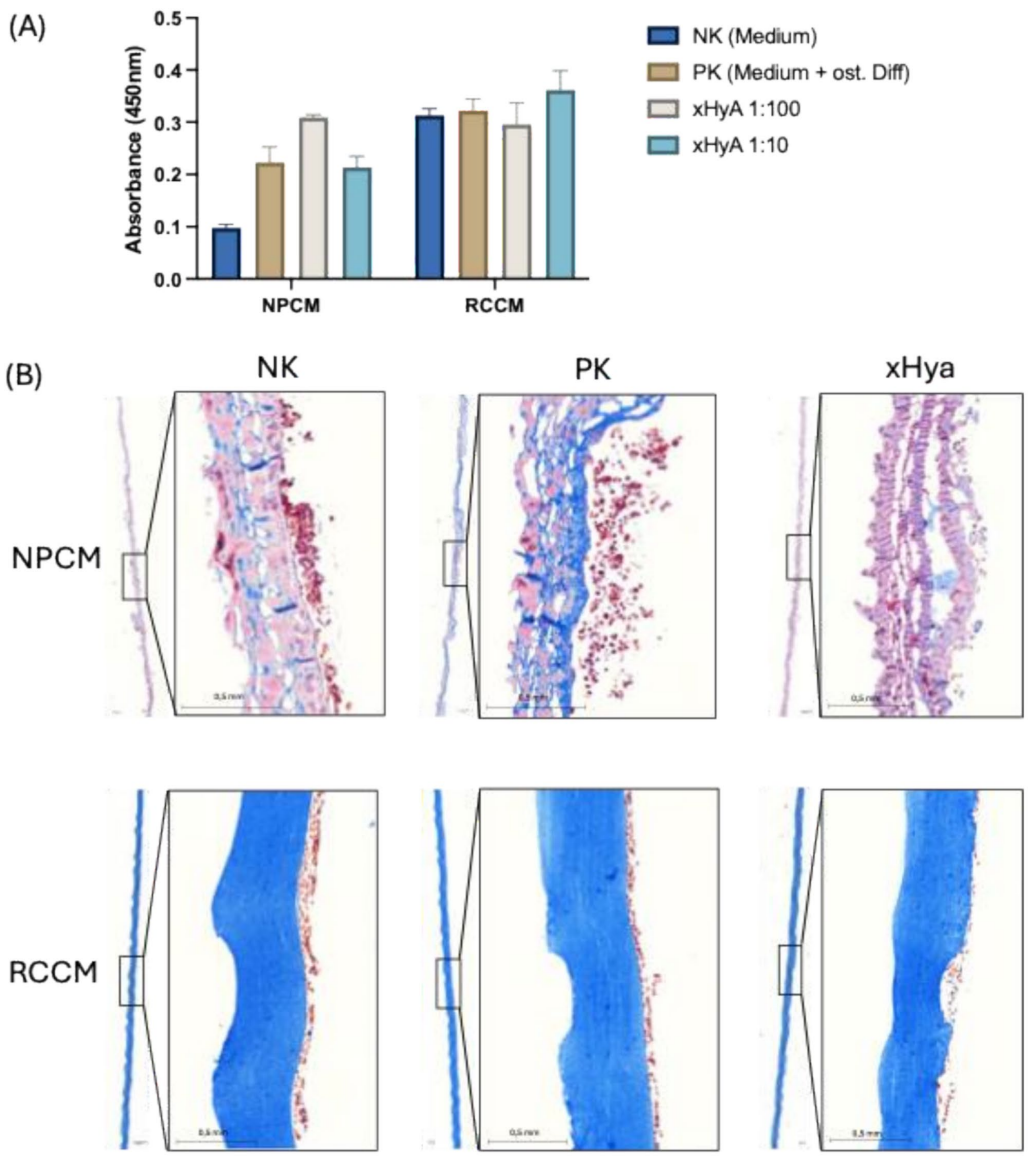
(**A**) Cell viability measurements on both substrates. The blank was measured with cell-free complete medium supplemented with WST-8. All samples were corrected by subtraction of the blank. (**B**) Histochemical analyses on exemplary substrates stained with Azan and Alcian Blue. NPCM = Native pericardium membrane, RCCM = ribose crosslinked collagen membrane, NK = negative control, PK = positive control supplemented with osteogenic medium, xHya = xHya supplemented osteogenic medium

Both substrates exhibited viable and proliferating cells in the airlift model, indicating that both membrane types show good cytocompatibility (Fig. 2A). However, the NPCM has a looser structure, which allows cells to penetrate the membrane, while the RCCM exhibits a very dense structure, with cells adhering on one side only. Surprisingly, we found xHyA remnants within the collagen scaffold of the NPCM (Fig. 2).

### xHyA increases alkaline phosphatase activity on collagen substrates

**Fig. 3:**
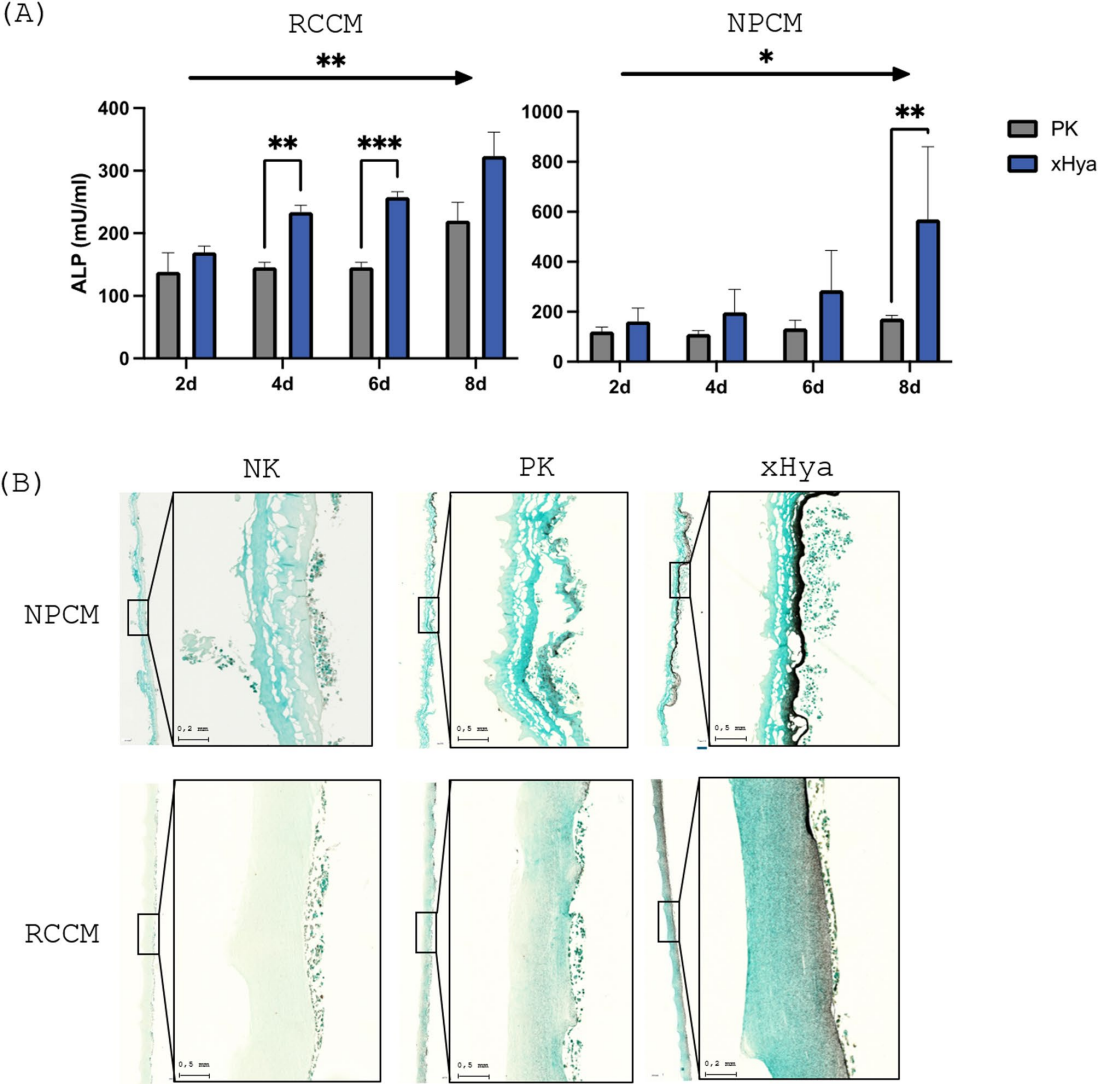
(**A**) ALP activity between positive control (PK) and xHya-pretreated groups (1:100) on different substrates. *=*p* < 0.05, **=*p* < 0.01, ***=*p* < 0.001. (**B**) Von Kossa staining of substrates indicating increased silver staining (dark grey) in xHya-pretreated groups

Unsurprisingly, both collagen substrates exhibited slightly increasing ALP activity within the first 8 days after seeding the SaOS-2 cells. Cells on the substrates functionalized with xHyA exhibited, however, significantly increased ALP activity (Fig. 3A) as early as 4 (RCCM) and 6 days after seeding (NPCM). After 8 days, the ALP activity remained significantly higher than in the untreated RCCM (*p* < 0.05) and NPCM (*p* < 0.001) accordingly. These findings were microscopically substantiated by Von Kossa staining, which revealed considerably increased silver precipitate on both substrates in response to xHyA treatment, respectively (Fig. 3B).

### xHyA promotes differential gene expression in a dose-dependent manner

**Fig. 4:**
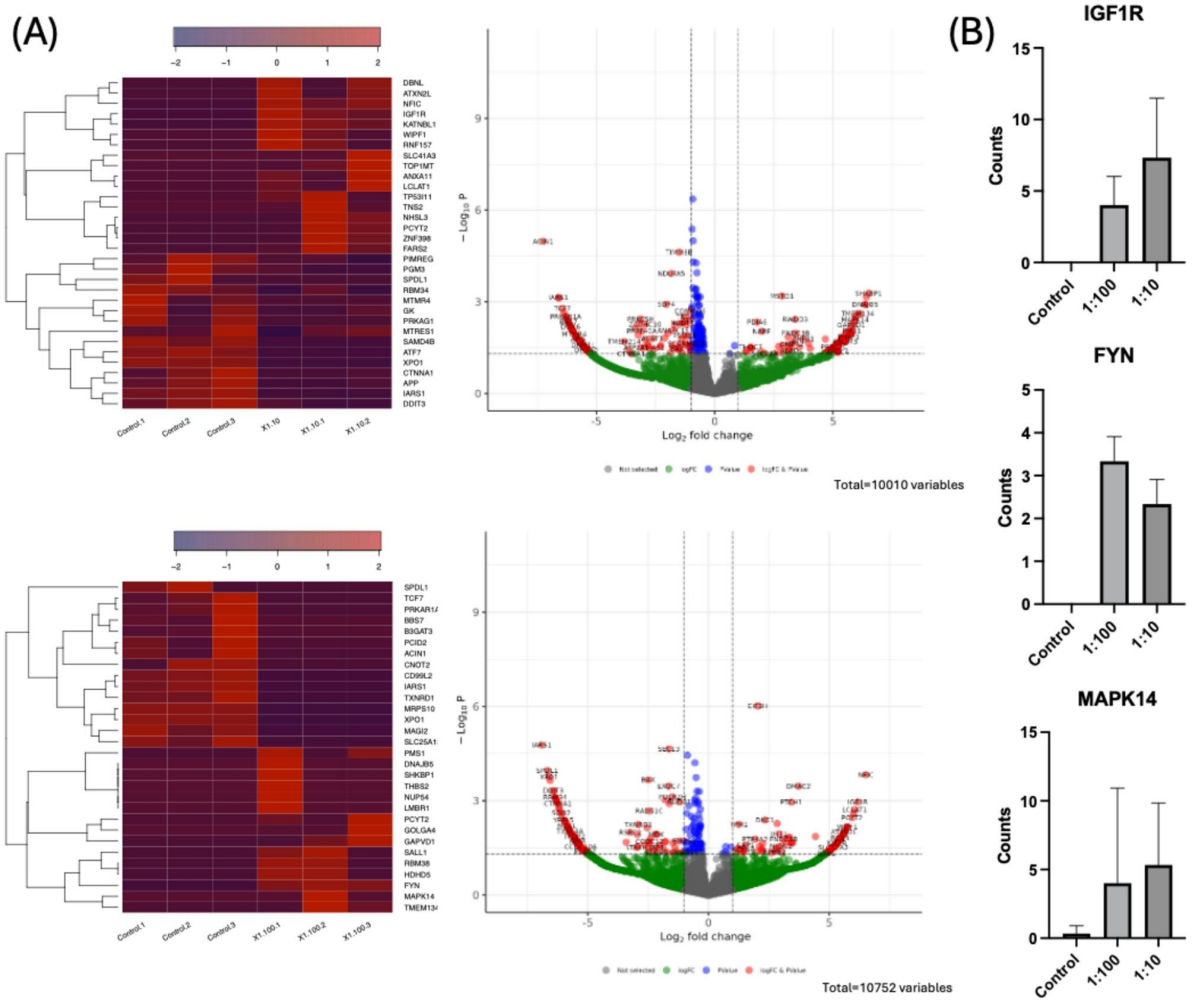
(**A**) Heatmaps and volcano plots of differentially expressed genes after treating SaOS-2 cells with xHya. The upper figures represent the higher concentration of xHya (1:10) and the lower figures represent the 1:100 dilution. (**B**) Dose-dependently upregulated genes IGF1R and FYN and upregulated gene MAPK14

**Fig. 5:**
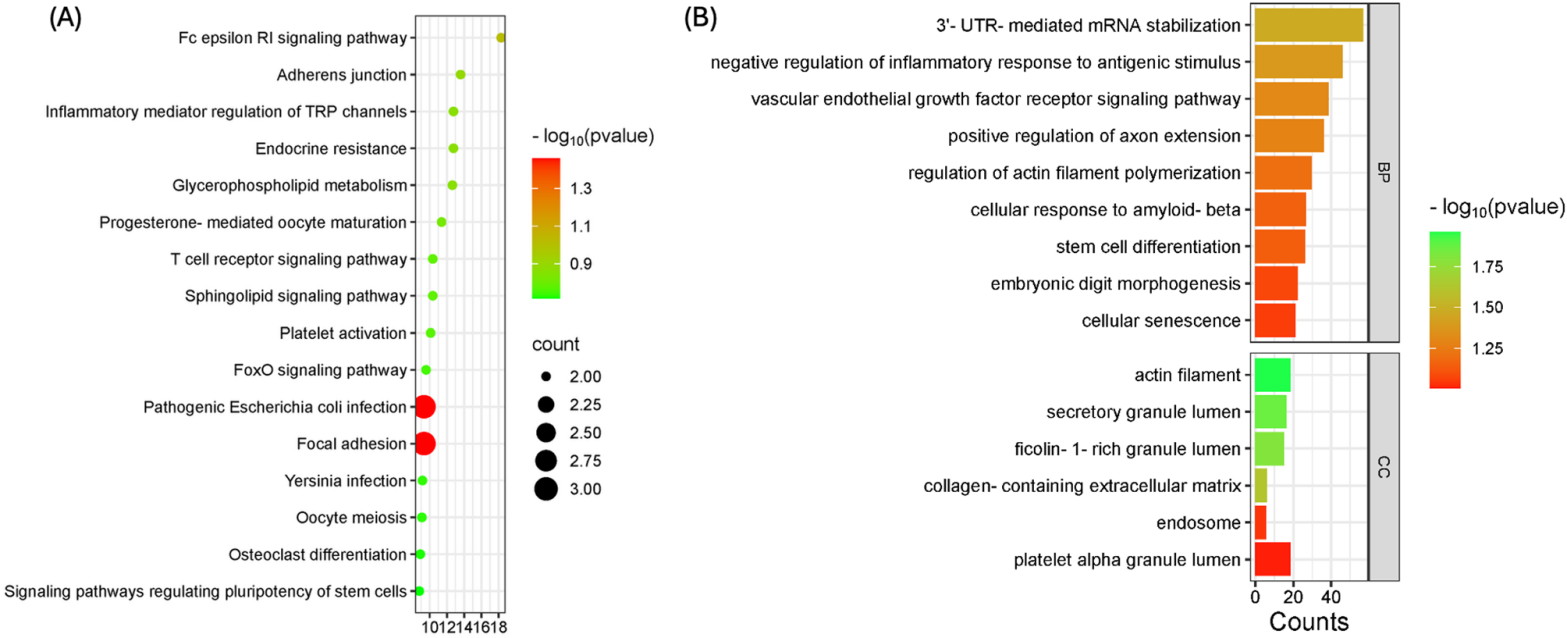
Pathway enrichment analysis for differentially expressed genes. (**A**) represents the KEGG pathway enrichment, figure (**B**) represents the Biological Process and the Cellular Component

To further evaluate the specific effects mediated by xHya, we compared the transcriptomes of untreated SaOS-2 cells with those from cells treated with xHyA-supplemented osteogenic medium (xHyA dilution of 1:10 and 1:100). A total of 1,126 pores were available on the flow cell, as indicated by the control software, before the sequencing run began. After 24 h of run time, 1,843,135 sequence reads were successfully aligned against the human reference genome GRCh38. The collagen substrates in this study were pretreated with different doses of xHyA. Surprisingly, RNA sequencing revealed different overexpressed genes in response to the varying concentrations (Fig. 4), except for IGF1R, FYN and MAPK14. Moreover, no genes encoding for bone-related proteins like ALP were significantly regulated, as indicated by the heat maps. The pathway enrichment analysis confirmed that xHyA has apparently no influence on bone-related gene expression in this experimental setting. However, the significantly overexpressed genes are overly related to, among others, focal adhesion and cell adherence, vascular endothelial growth factor signaling and stem cell differentiation (Fig. 5).

## Discussion

This study evaluated the osteogenic potential of BDDE-crosslinked hyaluronic acid (xHyA) on SaOS-2 cells cultured on ribose cross-linked collagen membranes (RCCM) and native pericardium collagen membranes (NPCM) in an airlift model. The findings demonstrate that xHyA enhances ALP activity and mineral deposition, although its effects on bone-related gene expression appear to be minimal.

Guided bone regeneration is considered the most clinically used and documented technique for local augmentation and defect restitution of deficient jawbone areas in conjunction with oral implant treatment [18, 19]. Membranes utilized in GBR must be biocompatible to integrate within the host tissues and exhibit suitable degradation kinetics and satisfactory physico-mechanical characteristics [20, 21]. Considering the principle of GBR, cytocompatibility is especially important to allow cells in the blood clot to adhere to the defect-facing membrane surface, facilitating proliferation and differentiation. In this study, both NPCM and RCCM supported cell viability and proliferation, indicating their potential suitability as membranes for GBR. However, structural differences between the two membranes affected cell distribution. The looser structure of NPCM permitted cell infiltration, whereas the denser structure of RCCM restricted cells to the surface (Fig. 2). The observed retention of xHyA residues in NPCM indicates that xHyA is not dissolved and washed out of the substrates but remains associated with the collagenous scaffold. Molecular dynamics studies have shown that collagen–HA interactions are stabilized by multiple hydrogen bonds and are influenced by proline hydroxylation [22]. In the present context, the retention of xHyA is likely related to hydrogen bonds forming between the hydroxyl groups of HA and the hydroxyl and amide groups of collagen, which help anchor the HA within the collagen scaffold. This notion is supported by the findings of Eliezer et al., who showed that collagen membranes adsorb most of the applied HA and release it gradually. In their study, 80% of HA was initially bound, with 61% retained after 15 min and 36% still present after 10 days, indicating a stable interaction with the collagen matrix and a controlled release [23].

Alkaline phosphatase (ALP) is a hydrolase widely expressed in most human tissues [24]. As an essential biomarker, ALP plays a pivotal role in bone mineralization by binding to bone matrix proteins and stimulating pyrophosphate hydrolysis, making it a renowned biomarker for bone formation [25–27]. In a series of in vitro experiments Stucki, et al. [25] showed that ALP expression and activity are both precursors to osteogenesis in guided bone regeneration in an organized connective tissue environment.

In this airlift model, we found a substantial increase in ALP activity during the first 10 days after seeding osteoblast-like cells onto the collagen substrates, consistent with the preclinical literature. Interestingly, the xHyA-treated substrates exhibited significantly higher ALP activity than the osteogenic positive control, suggesting that xHyA may enhance the initiation of osteogenic processes. Von Kossa staining corroborated these findings, indicating enhanced mineral deposition. These effects were consistent across both substrates; however, the substrate type influenced the degree of ALP activity and mineralization, possibly due to differences in hydroxylation as suggested above. Nonetheless, these findings align with prior research demonstrating that hyaluronic acid derivatives can enhance bone regeneration [9, 15].

Mathews et al. evaluated the osteogenic effects of various glycosaminoglycans on mesenchymal stem cells and found that hyaluronic acid mediated the most substantial upregulation of genes associated with osteoblast differentiation [28]. Zhang et al. substantiated these results, reporting that hyaluronic acid promotes osteogenic differentiation of human amniotic mesenchymal stem cells via the TGF-β/Smad pathway [14]. Surprisingly, the RNA sequencing performed in this study revealed minimal regulation of genes encoding for proteins indicative of osteogenic differentiation. This result may be method-related since we used RNA-Seq, while the abovementioned authors applied RT-qPCR, which constitutes a more sensitive assay [29]. However, the more probable cause of the deviating results is rooted in the model organism of choice. Our study used the SaOS-2 cell line, an osteosarcoma cell line with osteoblastic properties [30]. The cell line is known for extracellular matrix deposition and mineralization, making it suitable for bone-related experiments. However, since it represents a cancer cell with a distinct phenotype, it does not exhibit pluripotency like mesenchymal stem cells. Therefore, it constitutively expresses high amounts of osteoblast markers rather than upregulating them in response to a stimulus. This notion is underscored by He, et al. [31] who showed by immunofluorescence staining that SaOS-2 cells constitutively express high amounts of type 1 collagen, osteopontin, and osteocalcin. Within this frame of reference, it is reasonable to suggest that osteoblastic differentiation markers were not differentially expressed at a significant rate because the SaOS-2 cell line already expressed large amounts in both, the xHyA and the control group, respectively.

More importantly, these findings indicate that an additional mechanism likely underlies the observed xHyA-mediated effects on ALP activity and bone mineralization, as there was no significant upregulation of ALP transcripts. Instead, xHyA treatment influenced pathways associated with focal adhesion, cell adherence, vascular endothelial growth factor signaling and stem cell differentiation, as suggested by pathway enrichment of differentially expressed genes. Hyaluronic acid interacts with cell surface receptors such as CD44 and RHAMM, which activate intracellular signaling cascades associated with adhesion, proliferation, and differentiation [32–35]. These pathways are integral to cell-matrix interactions, suggesting that xHyA indirectly enhances osteogenesis by improving cellular adhesion and promoting a microenvironment conducive to proliferation.

The most pronounced and dose-independent upregulation was found for three genes, namely IGF1R, FYN, and MAPK14 (Fig. 4). The importance of Insulin-like growth factor 1 (IGF-1) in bone regeneration and maintenance has been established. IGF-1 plays a vital role in the differentiation of osteoprogenitor cells during this process [36, 37]. Disruption of the growth hormone/IGF-1 pathway in humans has been associated with delayed or unsuccessful healing of fractures [36]. Additionally, the role of IGF-1 signaling is essential for the mineralization of bone matrix, evidenced by an osteoblast-specific knockout of the IGF-1 receptor gene, IGF1R [38]. Considering this framework, the witnessed effects of xHyA in this study may be, among others, mediated through an upregulation of the IGF-1 receptor. A study from Reckenbeil et al. further highlights this notion, showing that IGF-1 positively affects cell proliferation and wound healing as well as osteoblast differentiation. Intriguingly, the authors also showed that IGF-1 binding led to phosphorylation of p38 mitogen-activated protein kinase (MAPK). MAPK14, another gene upregulated by xHyA in this study, codes for one of four p38 isoforms [39]. The p38 kinase is a critical signaling molecule that regulates cellular responses to stress and inflammation. It is activated by various signals, including environmental stressors, pro-inflammatory cytokines, and specific growth factors, and it plays a pivotal role in processes such as gene expression, cell differentiation, and bone homeostasis [40, 41]. Apart from the IGF-1 signaling pathway, p38 was also shown to be a downstream signaling molecule to CD44, underlining its possible role in the cellular response to xHyA [42, 43].

FYN, belonging to the Src family of kinases, plays a crucial role in various specialized signaling pathways, especially within the immune and nervous systems. It influences T-cell activation, integrin-mediated adhesion, and neuronal development by affecting cytoskeletal dynamics and cell signaling. Structural domains and post-translational modifications tightly regulate these kinases to maintain proper signaling function. FYN’s role in these pathways is essential for maintaining cellular communication and function in specific biological contexts [44]. While experimental evidence is scarce, it has been reported that FYN associates with CD44 in lymphocytes and promotes proliferation in osteoclast lineage cells [45, 46]. This indicates a possible role as a signaling molecule downstream to xHyA-binding CD44. However, further experimental evidence is warranted to elucidate the cellular pathways involved.

While the study demonstrates the regenerative potential of xHyA-functionalized collagen substrates, several limitations require consideration. First, the results should be validated in various model organisms, specifically primary osteoblast models. Moreover, phosphoproteomic analyses are required to confirm the involvement of the pathways observed in the transcriptome. Future studies with extended incubation periods should account for the long-term effects of xHya, focusing specifically on concentrations necessary in vivo. In this regard, more clinical studies and bedside-to-bench investigations are necessary to validate these findings. Additionally, since xHya seems to deliver effects independently from growth-factor related pathways, combinations with other osteoinductive agents appear worthy of further investigation.

Taken together, this study highlights the potential of xHyA-functionalized collagen substrates to enhance bone formation in vitro. By promoting ALP activity and mineral deposition without significantly altering bone-related gene expression, xHyA may act primarily through the modulation of cell-matrix interactions. These findings provide a foundation for further exploration of xHyA as a functional biomaterial for bone tissue engineering, emphasizing the importance of scaffold design and functionalization in optimizing osteogenic outcomes.

## Data Availability

No datasets were generated or analysed during the current study.
